# Making comparable measurements of bacterial respiration and production in the subtropical coastal waters

**DOI:** 10.1007/s42995-022-00133-2

**Published:** 2022-07-11

**Authors:** Cui Guo, Ying Ke, Bingzhang Chen, Shuwen Zhang, Hongbin Liu

**Affiliations:** 1 College of Marine Life Sciences, Institute of Evolution and Marine Biodiversity, Frontiers Science Center for Deep Ocean Multispheres and Earth System, Ocean University of China, Qingdao, 266003 China; 2 Division of Life Science, The Hong Kong University of Science and Technology, Hong Kong, SAR China; 3 Department of Mathematics and Statistics, University of Strathclyde, Glasgow, G1 1XH UK; 4 Hong Kong Branch of Southern Marine Science and Engineering Guangdong Laboratory (Guangzhou), Hong Kong University of Science and Technology, Hong Kong, SAR China; 5 College of Life Science, South China Normal University, Guangzhou, 510631 China; 6 Department of Ocean Science, Hong Kong University of Science and Technology, Hong Kong, SAR China

**Keywords:** Bacterial respiration, Bacterial growth efficiency, Bacterial production, Free-living bacteria, Winkler method

## Abstract

Accurate estimates of bacterial carbon metabolic rates are indispensable for understanding the regulation of carbon fluxes in aquatic environments. Here, changes in bacterial growth, production, and cell volume in both pre-filtered and unfiltered seawater during 24 h incubation were monitored. The methodological artifacts during Winkler bacterial respiration (BR) measurements in subtropical Hong Kong coastal waters were assessed. Bacterial abundance increased by 3- and 1.8-fold in the pre-filtered and unfiltered seawater after incubation, respectively. Bacterial production (BP) and cell volume also showed significant enhancement. Compared with the BR measurements obtained by the Winkler method, the instantaneous free-living BR measurements, after correction, decreased by ~ 70%. The time-integrated free-living BR and BP during 24 h incubation in the pre-filtered sample provided an improved estimate of bacterial growth efficiency, which increased by ~ 52% compared to the common estimations using the noncomparable measurements of integrated free-living BR and instantaneous total BP. The overestimation of BR also exaggerated the contribution of bacteria to community respiration, affecting the understanding on the metabolic state of the marine ecosystems. Furthermore, the BR estimates by the Winkler method may be more biased in environments with a higher bacterial growth rate and tightly coupled grazing mortality, as well as in those with higher nutrient concentrations. These results reveal obvious problems associated with the BR methodology and raise a warning for caution when comparing BP and BR, as well as when making estimations of carbon flow through the complex microbial networks in aquatic ecosystems.

**Supplementary Information:**

The online version contains supplementary material available at 10.1007/s42995-022-00133-2.

## Introduction

Heterotrophic bacteria are one of the most abundant biotic components in aquatic systems and are the major mineralizers of aquatic organic carbon—the largest organic carbon reservoir on earth (Azam and Malfatti 2007). Bacterial respiration (BR) quantifies the amount of organic carbon respired back to inorganic carbon, and it is therefore a key parameter for understanding the role of bacteria in carbon remineralization (Robinson 2008). Simultaneous measurements of BR and bacterial production (BP) allow the estimation of another key parameter, bacterial growth efficiency (BGE), i.e., BP/(BP + BR), which describes the proportion of total assimilated carbon that is allocated to the synthesis of bacterial biomass. The accuracy of BR and BGE estimations fundamentally affect our understanding of carbon flow through the bacterial compartment and how bacteria regulate the biogeochemical state of the waters.

The most widely used method for BR measurement is the Winkler oxygen consumption method with bottle incubations to measure oxygen consumption of a 0.6–3 μm pre-filtered seawater sample undergoing an in vitro dark incubation for ~ 24 h (Robinson and Williams 2005). However, the prefiltration procedure and the subsequent bottle incubation used in this method give rise to various problems. First, the physical separation of cells in different size fractions breaks the trophic linkages between bacteria and their predators, as well as the interactions between bacteria and their autotrophic competitors. Removal of phytoplankton and their organic excretion, the leakage of cytoplasmic inclusions by the physical force during the filtration and the changes in microbial community structures may also alter substrate availability in filtered samples (Gattuso et al. 2002; Massana et al. 2001; Pomeroy et al. 1994; Sherr et al. 1999). The combined effects of filtration, including relief of grazing pressure and phytoplankton competition, and enrichment of inorganic and organic nutrients, may cause an increase in bacterial abundance at the end of the incubation, leading to a potential overestimation of the real BR (Aranguren-Gassis et al. 2012; Martínez-García et al. 2013; Pomeroy et al. 1994; Robinson 2008; Sherr et al. 1999). The average cell-specific BR (sBR), which depends mainly on cell size and temperature, may also increase as some studies have shown that long-term incubation after filtration favors the growth of bigger bacteria with high DNA content (Gasol and Morán 1999; Massana et al. 2001; Martínez-García et al. 2013). Indeed, the reported BR sometimes contributed > 90% of total community respiration (CR) in oligotrophic waters and could even occasionally exceed CR or primary production (Aranguren-Gassis et al. 2012; Biddanda et al. 2001; Kirchman et al. 2009). These exceptionally high BR rates are possibly artifacts created by the Winkler oxygen consumption method. This overestimation of BR may also affect the estimation of metabolic balance between respiration and primary production in the oligotrophic ocean, which is a long-standing scientific controversy (Duarte et al. 2013; Williams et al. 2013). Second, the noncomparable incubation time frames and size ranges of the microbial communities included in BP and BR measurements may cause inaccurate estimates of BGE. For example, while BR is typically derived from a 24 h (or longer) incubation of filtered water samples with only free-living (FL) bacteria, BP is measured almost instantaneously (i.e., within 30 min to 3 h) in unfiltered water samples including all bacterial components. Inaccurate estimations of BGE directly affects any subsequent evaluations of carbon flow through the microbial loop.

Although methodological problems have already been raised in some studies (Aranguren-Gassis et al. 2012; Pomeroy et al. 1994), how these experimental artifacts affect BR estimates have been rarely assessed and few solutions has been proposed to improve this method. Instead, a different method quantifying the in vivo respiratory electron transport system (ETS) activity was developed to infer cellular respiration in marine microbial planktonic communities (Martínez-García et al. 2009). The ETS method measures the formation rate of insoluble formazan crystals trapped in cells by the reduction of a membrane-permeable tetrazolium salt, 2-(4-iodophenyl)-3-(4-nitrophenyl)-5-(phenyl) tetrazolium chloride (INT). The relatively higher sensitivity of the ETS method reduces the bottle incubation time to several hours and size-fractionated respiration by the bacterial communities is obtained by a post-incubation filtration step. This method, which has been successfully employed in field studies has detected potential overestimation of BR obtained by the Winkler method (Aranguren-Gassis et al. 2012; García-Martín et al. 2019; Martínez-García et al. 2013). However, accumulation of the toxic formazan crystals in cells might be fatal to living microorganisms, and eukaryotes and prokaryotes may reduce the substrate, INT, at different efficiencies (Baños et al. 2020; Villegas-Mendoza et al. 2015). Some indirect approaches estimate BR based on predictions from measured BP or temperature (del Giorgio and Cole 1998; López-Urrutia and Morán 2007; Robinson 2008). These empirical models were established with published BR rates that were mostly measured by the Winkler method. Considering the potential overestimation of these BR rates, the coefficients of these models may need to be recomputed with corrected data sets.

**Fig. 1:**
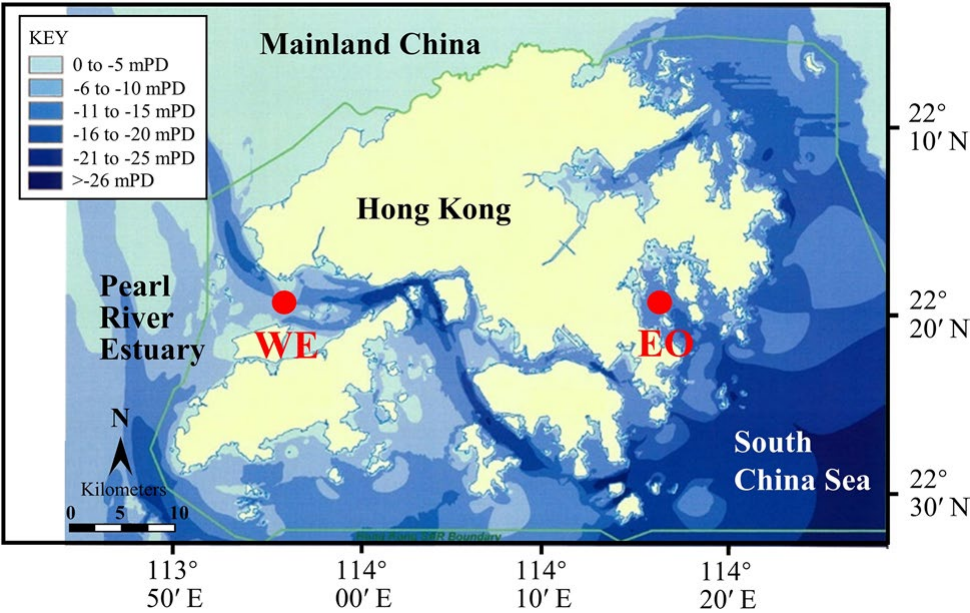
Locations of the EO (Eastern Oceanic) and WE (Western Estuarine) sampling stations

In the present study, the methodological biases due to prefiltration and incubation in the classic Winkler BR measurement were evaluated by conducting monthly sampling at two contrasting coastal sites, a western estuarine station (WE, 22° 21.32′ N, 113° 56.78′ E) and a relatively pristine eastern oceanic station (EO, 22° 20.45′ N, 114° 17.70′ E), in Hong Kong coastal waters in the NW Pacific with a distinct wet season from May to October and a dry season, from November to April, from November 2014 to October 2015 (Fig. 1). EO station is mainly influenced by coastal waters of the South China Sea, whereas WE station is strongly affected by the nutrient-rich discharge from the Pearl River. Bacterial abundance, production and cell volume were measured before and after incubation in both pre-filtered (for BR measurement) and unfiltered (for CR measurement) seawater samples. Instantaneous BR rates were estimated by removing the experimental artifacts induced by increase of bacterial biomass at the end of the incubation in the filtered samples in the Winkler oxygen consumption method. Meanwhile, by attaining both integrated mean BR and BP during 24 h incubation in the filtered sample, an improved BGE was obtained. The corrected BR and BGE were compared to those before correction and the degree of overestimation of BR and underestimation of BGE were evaluated at the two sites.

## Results

### Ambient hydrographic conditions of WE and EO sites

**Fig. 2:**
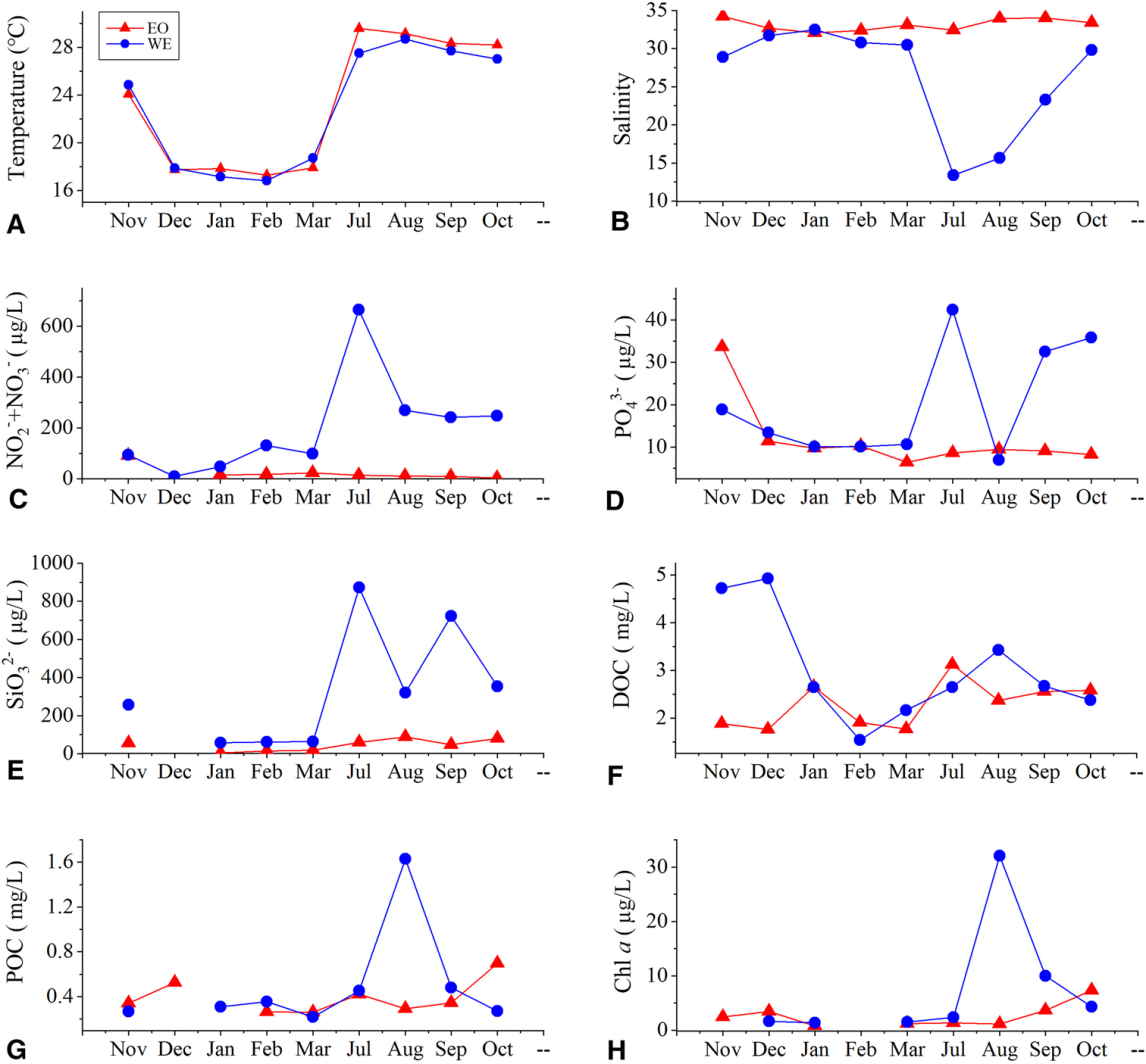
Hydrographic conditions of the WE and EO sampling stations. Parameters measured include **A** temperature, **B** salinity, **C** nitrite and nitrate (NO_2_^−^ + NO_3_^−^), **D** phosphate (PO_4_^3−^), **E** silicate (SiO_3_^2−^), **F** dissolved organic carbon (DOC), **G** particulate organic carbon (POC) and (H) chlorophyll *a* (Chl *a*)

The temperature at the two stations was similar throughout the year, with mean values of ~ 19 °C in the dry season and ~ 28 °C in the wet season (Fig. 2A). At EO station, the salinity was consistent (at ~ 33) throughout the year. In contrast, at WE station, the salinity dropped to ~ 21 in the wet season due to the Pearl River discharge (Fig. 2B). The Pearl River discharge also resulted in significantly higher concentrations of NO_2_^−^ + NO_3_^−^, PO_4_^3−^ and SiO_3_^2−^ at WE than at EO, especially in the wet season (Fig. 2C–E). This enhanced nutrient loading in WE during the wet season stimulated the growth of phytoplankton, as reflected by the higher concentrations of Chl *a* and particulate organic carbon (POC) from July to September (Fig. 2G, H). At EO, however, the concentrations of Chl *a* and POC showed no significant differences between the two seasons (Fig. 2G, H). The concentration of dissolved organic carbon (DOC) was higher at WE station in the dry season, but similar between WE and EO in the wet season (Fig. 2F).

## Change in bacterial abundance, cell size and growth rate in pre-filtered and filtered seawater during BR incubation

**Fig. 3:**
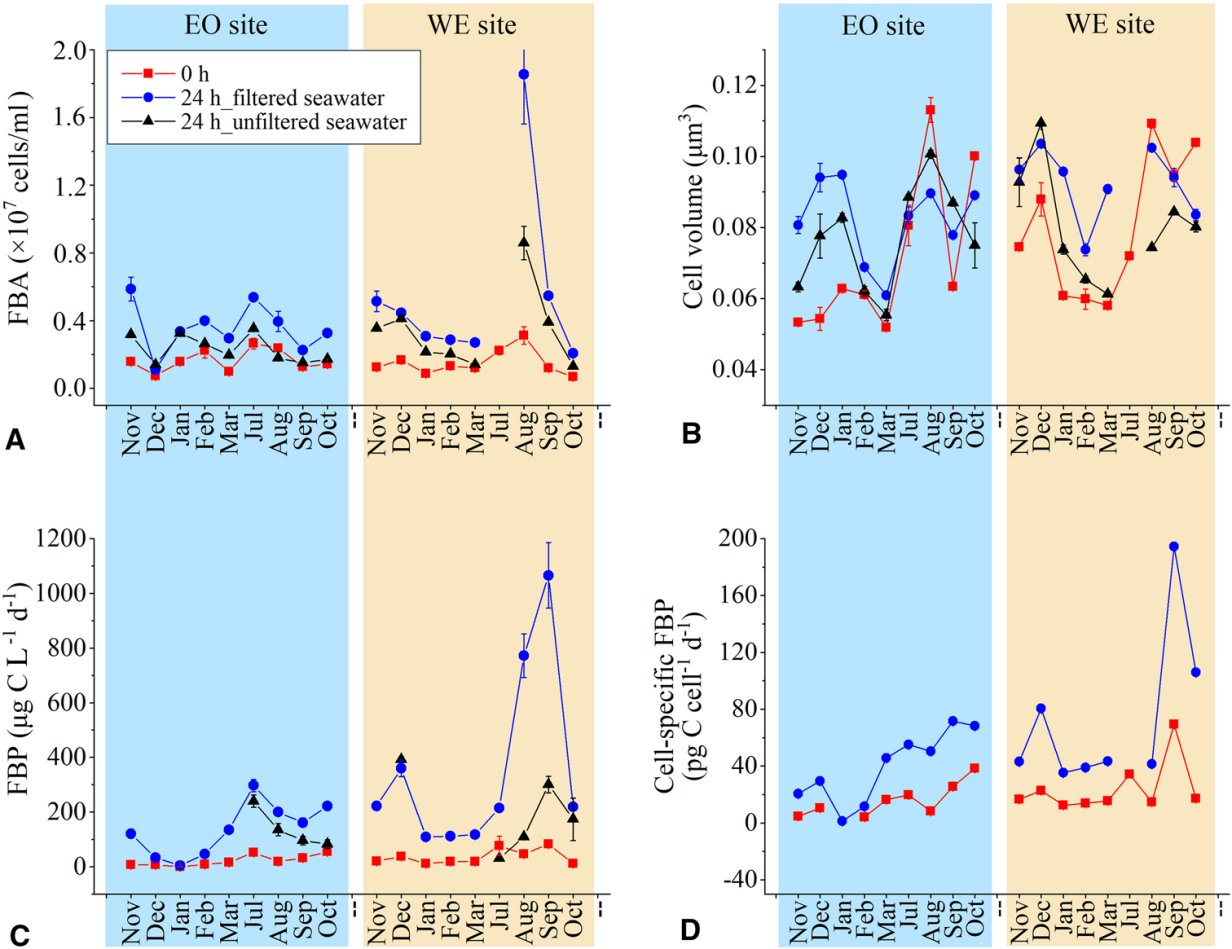
Comparison of free-living **A** bacterial abundance (FBA), **B** cell volume, **C** bacterial production (FBP) and **D** cell-specific FBP at the initial time point and after 24 h incubation in the pre-filtered and unfiltered seawater samples at an eastern oceanic (EO) and a western estuarine (WE) site in NW Pacific. Error bars represent standard deviation of replicates or propagated errors from the measurements

The average free-living bacterial abundance (FBA) was similar at the two sampling sites (1.7 × 10^6^ cells/ml at EO and 1.5 × 10^6^ cells/ml at EO) and was higher in the wet season (1.9 × 10^6^ cells/ml) than in the dry season (1.3 × 10^6^ cells/ml) (Fig. 3A). In the pre-filtered seawater, the 24 h incubation led to a 2.2- and 3.7-fold increase at EO and WE, respectively. A smaller increase of 1.4- and 2.2-fold was obtained in the fraction of FL bacteria in the unfiltered seawater.

Cell size of FL bacteria was slightly larger at WE station (~ 0.076 μm^3^) than at EO station (~ 0.061 μm^3^) (Fig. 3B). After 24 h incubation, however, cell volume of FL bacteria increased significantly by ~ 30% in the pre-filtered seawater (Paired t-test, *n* = 18, *P* < 0.01). A slight increase of FL bacterial cell size by ~ 12% in the unfiltered seawater was also observed after the incubation. Growth rate was thus estimated by the accumulation of cell volume-normalized carbon biomass instead of bacterial abundance as we described in the methods.

**Fig. 4:**
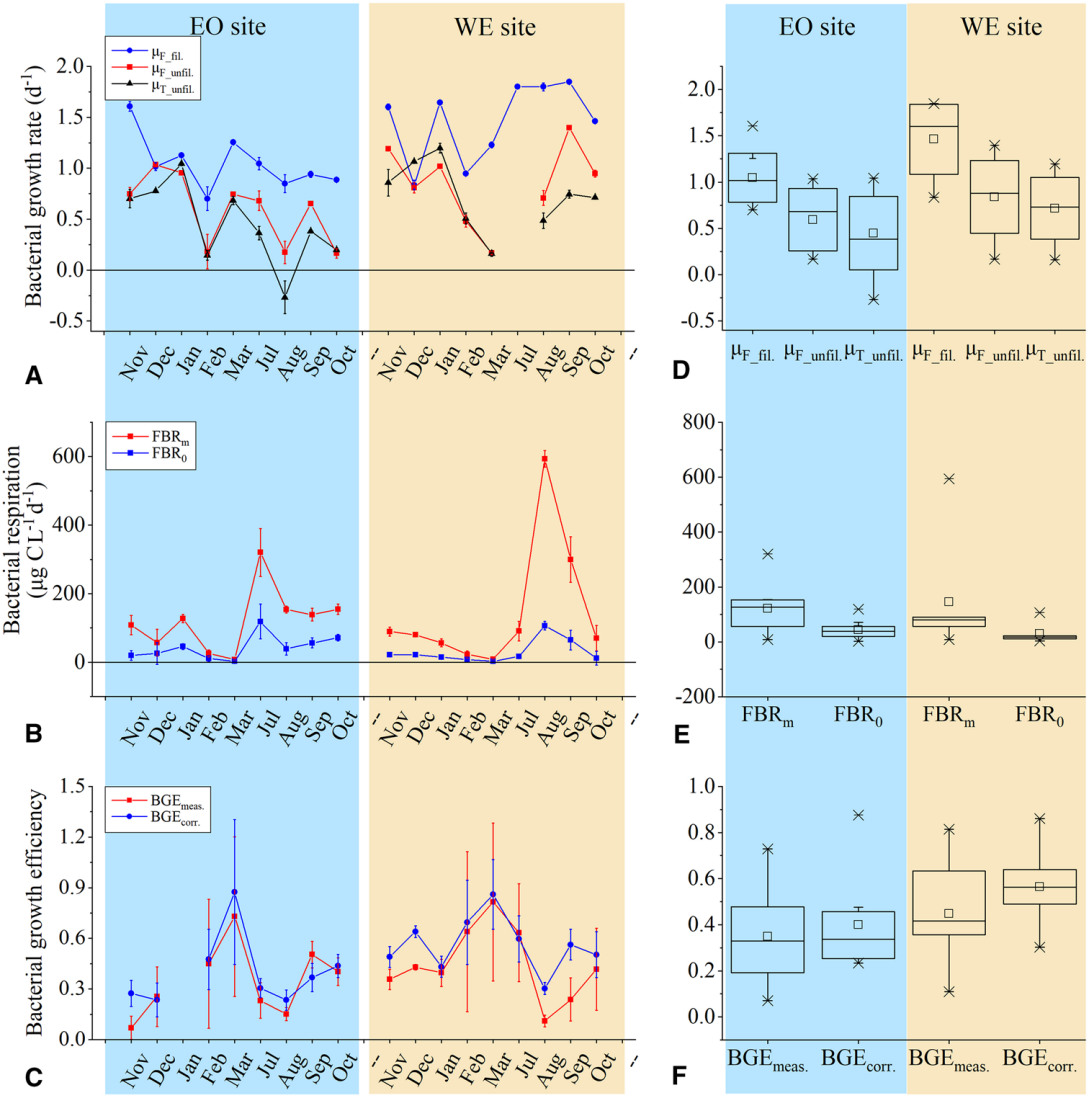
Comparison of bacterial **A**, **D** growth rate (*μ*), **B**, **E** respiration rate and **C**, **F** growth efficiency before and after correction. The data are present as both individual datasets **A**–**C** obtained monthly and boxplots **D**–**F** showing the distribution of values at EO and WE site. Error bars represent standard deviation of replicates or propagated errors from the measurements. Abbreviations of metabolic parameters are described in Table 1

**Fig. 5:**
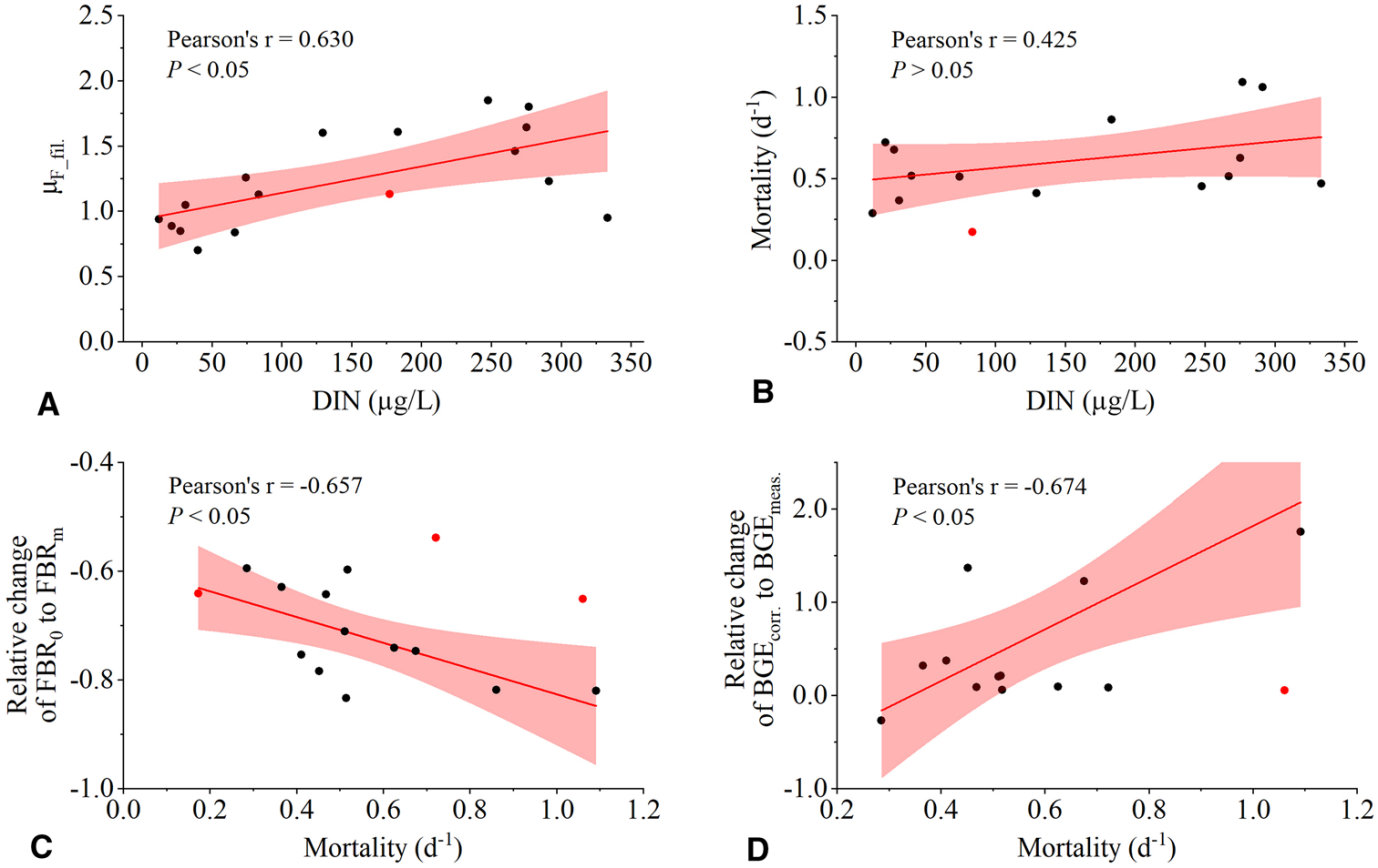
Linear regression analysis among bacterial gross growth rate, grazing mortality, relative changes of bacterial respiration and growth efficiency, and the concentration of dissolved inorganic nitrogen (DIN). Bacterial grazing mortality was estimated by the difference between bacterial growth rate in pre-filtered and unfiltered seawater samples. The r and *P* values are coefficients of Pearson’s correlation analysis and statistical significance levels. Abbreviations of metabolic parameters are described in Table 1

Growth rate of FL bacteria in unfiltered seawater samples (*μ*_F_unfil._) was 0.59 day^−1^ (ranging from 0.17 to 1.03 day^−1^) at the EO station, lower than the rate of 0.84 day^−1^ (ranging from 0.17 to 1.40 day^−1^) measured at the WE station (Fig. 4A, D). After removing bacterial grazers and other larger particles in 1 μm filtered seawater, growth rate of FL bacteria (*μ*_F_fil._) was significantly enhanced (paired *t*-test, *n* = 17, *P* < 0.01), ranging from 0.70 to 1.60 day^−1^ at the EO station and 0.84–1.85 day^−1^ at the WE station, respectively (Fig. 4A, D). It exhibited a significant positive correlation with the dissolved inorganic nitrogen concentration (Fig. 5A). Overall, *μ*_F_fil._ was 2.4-fold higher than *μ*_F_unfil._ (paired *t* test, *n* = 17, *P* < 0.01). Compared with the net growth rate of total bacteria in the unfiltered samples (*μ*_T_unfil._, 0.15–1.20 day^−1^), *μ*_F_fil._ was also significantly higher by 2.3-fold (paired *t* test, *n* = 17, *P* < 0.01), confirming that bacterial growth was stimulated in the pre-filtered seawater samples and that there was a substantial accumulation of bacterial biomass over the time course of the incubation.

### Change in BP in pre-filtered and filtered seawater during BR incubation

The initial free-living bacterial production (FBP) ranged from 7.4 to 83.2 μg C L^−1^ day^−1^ with an average value of 31 μg C L^−1^ day^−1^. This demonstrated similar spatial and seasonal patterns to FBA (Fig. 3C). It was significantly higher at WE (36.1 μg C L^−1^ day^−1^ on average) than that at EO (25.1 μg C L^−1^ day^−1^), and in the wet season (47.3 μg C L^−1^ day^−1^) than in the dry season (16.5 μg C L^−1^ day^−1^) (paired *t* test, *P* < 0.01). After incubation, it increased dramatically by 5.4- and 9.8-fold at EO and WE in the pre-filtered seawater, respectively. The FBP in the unfiltered seawater after incubation was also measured from July to October and exhibited smaller increases compared with those in the pre-filtered seawater.

The degree of increase of bulk FBP was larger than that of FBA, leading to the observation of increases in the cell-specific FBP (sFBP, Fig. 3D). Compared with the initial sFBP, incubation resulted in a 2.5- and 3.0-fold enhancement at EO and WE, respectively.

### Comparison of BR and BGE before and after correction

With a substantial accumulation of bacterial biomass and production in pre-filtered samples, BR measured by the Winkler method as an integrated mean rate of FL BR over the 24 h incubation (FBR_m_), was expected to be higher than the instantaneous initial BR (FBR_0_) and thereby was overestimated. It varied from 8 to 320 μg C L^−1^ day^−1^ with a mean value of 121 μg C L^−1^ day^−1^ at the EO station and 9–593 μg C L^−1^ day^−1^ with a mean value of 146 μg C L^−1^ day^−1^ at the WE station (Fig. 4B, E). After correction, the instantaneous FBR_0_ was much lower in all measurements, ranging from 2 to 106 μg C L^−1^ day^−1^ with mean values of 43 and 30 μg C L^−1^ day^−1^ at the EO and WE station, respectively (Fig. 4B, E). For the two extraordinarily high values (outliners in Fig. 2E) obtained in Aug at WE and Jul at EO in particular, the correction reduced the values by 82% and 63%, respectively. Compared to FBR_m_, the corrected FBR_0_ decreased by 75% (± 7%, 1 standard deviation) at WE and 65% at EO (± 9%, 1 standard deviation). Thus, the Winkler oxygen consumption method overestimated in situ FBR_0_ by ~ 209% and 332% at EO and WE, respectively. The degree of the negative relative change of FBR_0_ to FBR_m_ was significantly correlated with the top–down mortality that was estimated as the difference in bacterial growth rate between pre-filtered and unfiltered seawaters (Fig. 5C).

In most studies, BGE (BGE_meas._) has been obtained from direct measurements of BR by the Winkler method (FBR_m_) and instantaneous total bacterial production at the initial time point (TBP_0_). However, as TBP_0_ and FBR_m_ were not matched in terms of incubation time and bacterial-size scale, BGE_meas._ may deviate from in situ values (BGE_corr_) calculated by integrated mean BP (FBP_m_) and BR (FBR_m_) measured in parallel with the BR bottles during incubation (Fig. 4C, F). The mean BGE_meas._ was 0.35 and 0.45 at EO and WE, respectively. Using FBR_m_ and FBP_m_, the corrected BGE_corr._ was 0.41 and 0.56 at EO and WE, which increased significantly by ~ 52% relative to corresponding BGE_meas._ on average (Fig. 4C, F). Generally, the real BGE_corr._ was under-estimated by ~ 21% compared to BGE_meas_.

### Contribution of BR to CR (BR%)

**Fig. 6:**
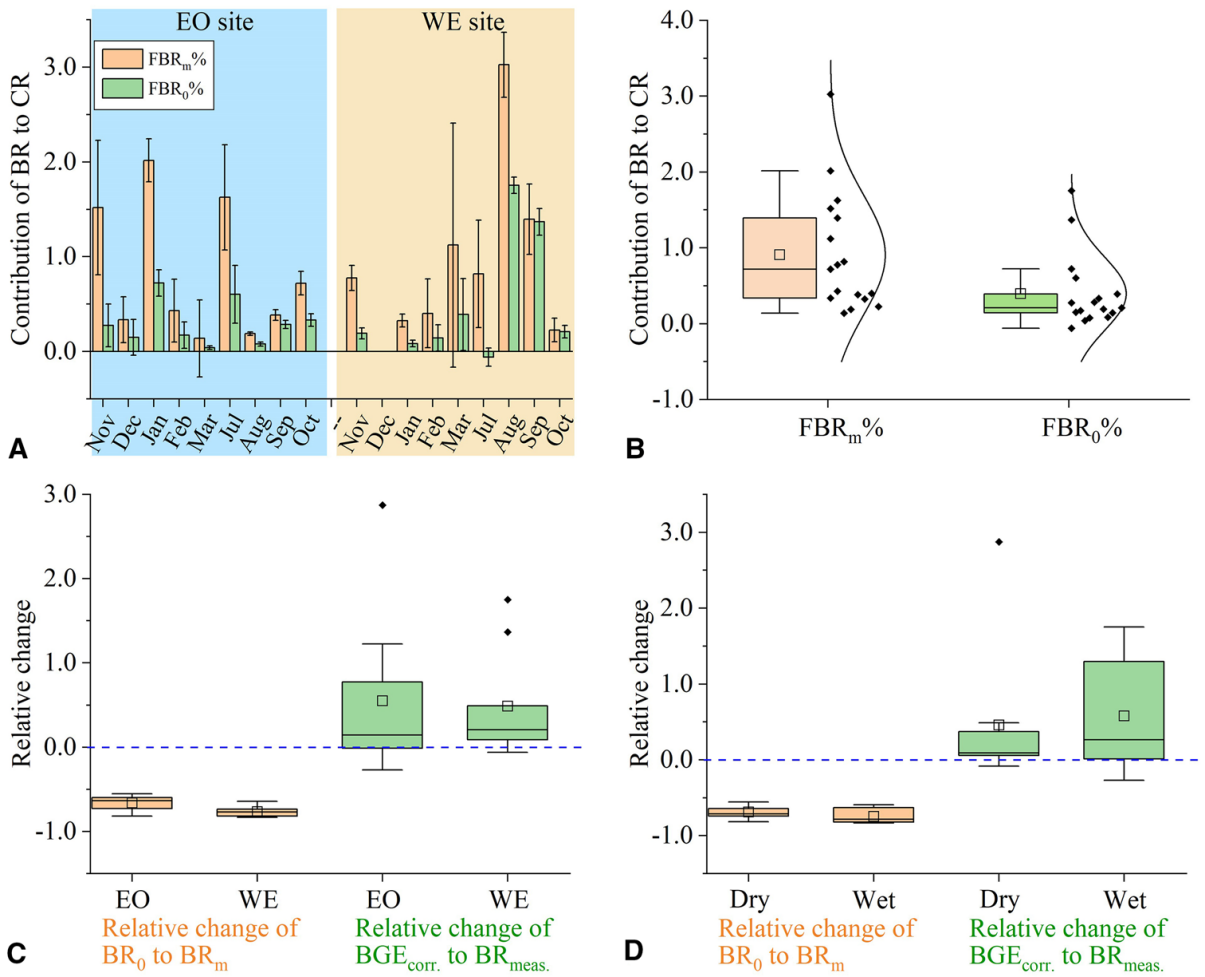
**A**, **B** Comparison of the contribution of free-living bacterial respiration (FBR) to the community respiration rate (CR) before (FBR_m_%) and after correction (FBR_0_%). The data are present as both individual datasets **A** obtained monthly and boxplots **B** showing the distribution of values at EO and WE sites. **C**, **D** Box plots showing relative changes of corrected **C** free-living bacterial respiration (FBR_0_) and **D** growth efficiency (BGE_corr._) compared to the direct determination by the Winkler method (FBR_m_, BGE_meas._) at EO and WE in dry and wet seasons, respectively. Relative change is calculated by (*A* − *B*)/*B*, in which *A* is the corrected value and *B* is the value before correction

The FBR_m_ measured by Winkler method accounted for ~ 91% in CR on average, ranging from 14 to 300% (Fig. 6A, B). In particular, the average contribution reached 100% at WE and was 82% at EO, with six out of eighteen experiments exceeding 100%. However, using FBR_0_, the contribution of BR decreased significantly to 26% on average (Paired t test, *n* = 17, *P* < 0.01).

## Discussion

### Methodological considerations

**Fig. 7:**
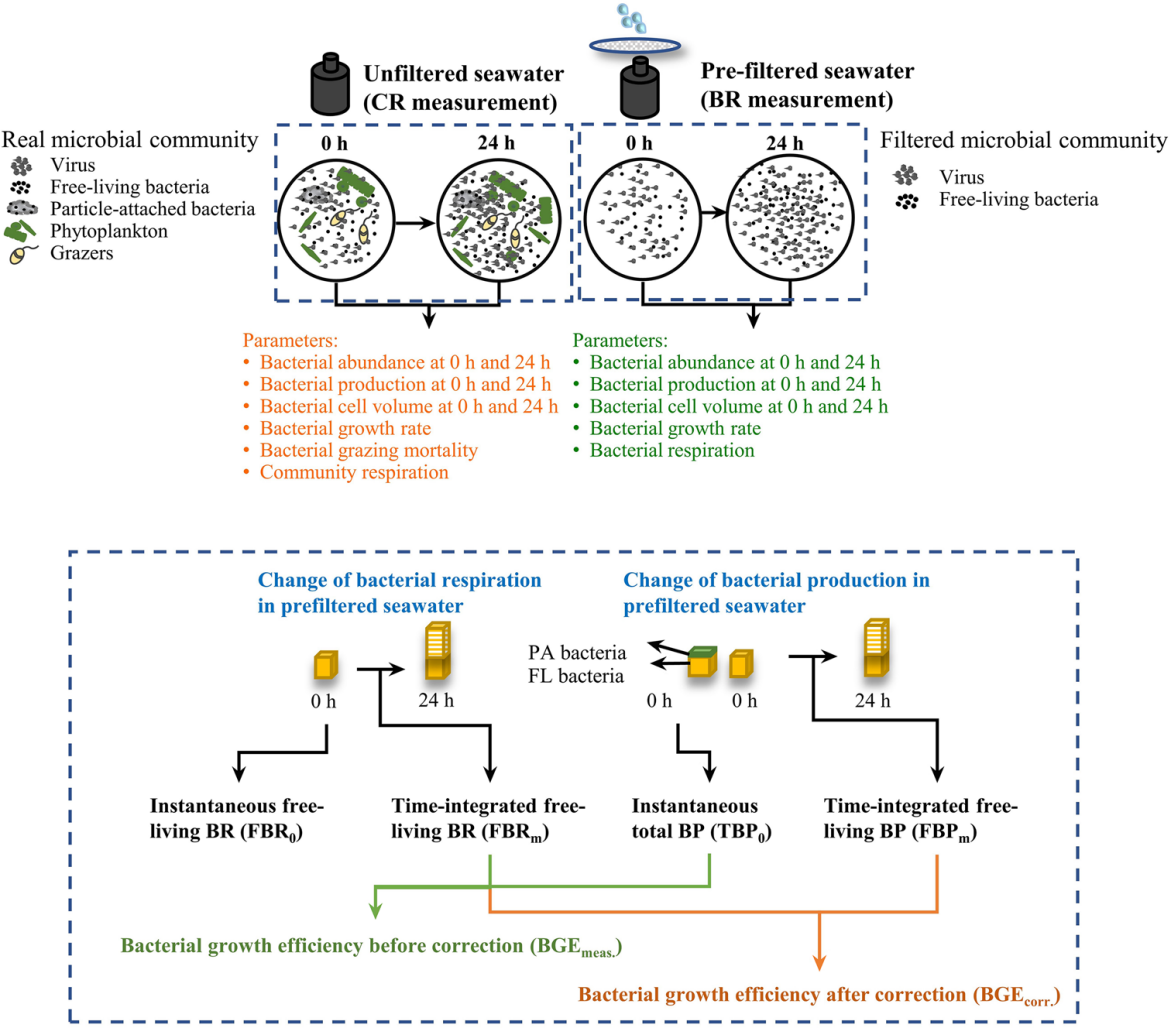
Schematic diagram showing the artifacts and potential overestimation of bacterial respiration measured by the incubation-based oxygen consumption Winkler method

This study corrects FBR_m_ measured by the Winkler oxygen consumption method by calculating an instantaneous FBR_0_ at the initial time point to minimize experimental artifacts created by filtration and bottle incubation procedures (Fig. 7). The correction for FBR was performed by assuming 1) the change in bacterial carbon biomass and production in filtered seawater samples with time followed an exponential model during the incubation (Eq. 1 in the Method section); 2) the carbon biomass-normalized (volumetric) BR was constant over the time course of the incubation. The exponential growth of bacteria was confirmed by time series sampling of bacterial biomass at EO and WE (Liu et al. in prep.; Supplementary Fig. S1) and in previous studies (Pomeroy et al. 1994). This model has also been widely used in lab and field studies for calculating bacterial growth rate (Kirchman 1982; Simon and Azam 1989; Ducklow et al. 1999). Compared with variable BP that highly depends on nutrient availability, cell-specific BR is mainly dependent on temperature and less affected by nutrients (Apple et al. 2006; López-Urrutia and Morán 2007). Thus, in nutrient-enriched coastal regions, the biomass-normalized BR should be relatively stable and would not be much affected by the nutrient fluctuation induced by filtration. Moreover, the correction for BR of FL bacteria in filtered samples already considered the influence of changes in nutrients, which is one of the prefiltration-induced factors causing the increase in bacterial biomass at the end of the incubation in filtered samples.

The accuracy of BGE estimates was only evaluated with the corrected BR. The potential problems in BP measurements and its influences on BGE estimates were not assessed in this study. Values of respiratory quotient (RQ) and leucine conversion factor may also affect the accuracy of BR and BP and need to be empirically determined for different ecosystems (Berggren et al. 2012; del Giorgio et al. 2011). Here, it was not the intention to solve all the uncertainties that prevent the accurate estimates of BR in the field nor to deny or change the existing experimental method. By providing an improved analysis of the data, our results shed light on potential problems of the current oxygen consumption BR measurement method. Our data clearly show that bacterial abundance, biomass and production increase dramatically, and the BR of FL bacteria were significantly overestimated in filtered samples after incubation. These results enable a better understanding of how the Winkler oxygen consumption method fails to provide an accurate estimation of the real BR and its influence on subsequent estimations of other carbon metabolic parameters that depends on BR.

### Potential overestimation of instantaneous BR and the contribution of BR to CR

Filters of 1 µm pore diameter were chosen for obtaining FL bacteria; they retained ~ 88.6% of total heterotrophic bacteria and 53.4% of total BP in filtered samples (data not shown). This is comparable to the results of Del Giorgio et al. (2011) who reported on the use of a glass fiber filter with a pore-size of 1.2 µm, which had a maximum retention efficiency of 84% of heterotrophic bacteria in filtered samples and Martínez-García et al. (2013) who showed a ~ 50% reduction of BP after 0.8-µm filtration.

Large increases in bacterial abundance and production were observed in the pre-filtered seawater of the BR incubations, due to relief from grazing pressure and a change in the substrate content; this was consistent with previous studies (Gasol and Morán 1999; Gattuso et al. 2002; Massana et al. 2001; Pomeroy et al. 1994; Sherr et al. 1999). Compared with the response in the pre-filtered seawater, smaller increases were obtained in the unfiltered seawater after incubation, indicating that a stimulation of FL bacterial growth also occurred in CR measurement. FL bacterial cell volume also increased, suggesting that complex responses of the FL bacterial community occurred in the bottle during the BR and CR measurements. This is possible due to the metabolic shift up, as the degree of increase in BP (~ ninefold) was much higher than that of BA (~ threefold). Community composition change and selective grazing might also be the reasons for these increases (Massana et al. 2001; Pomeroy et al. 1994; Sherr et al. 1999). Indeed, clear bacterial community structure change was observed during the BR and CR incubations at both EO and WE (Guo et al. in prep.).

All the above changes in the bacterial community, especially the stimulated growth of bacteria compared with the in situ conditions, led to the overestimation of incubation- and prefiltration-based BR measurements. To eliminate these influences, an improved BR of FL bacteria was estimated with a more accurate BGE that was calculated by the unified BP and BR in terms of bacterial-size scale and incubation time (discussed in the following section) and an in situ BP of FL bacteria measured at the initial time point (FBP_0_). Such corrections allow an estimation of an instantaneous FBR_0_ that minimizes the influences of filtration and long-time incubation. A mean overestimation of ~ 270% in BR measured by the pre-filtered incubation Winkler method was obtained from the coastal Hong Kong waters in this study. This value coincided with those of Martínez-García et al. (2013) who reported an overestimation of ~ 264% in pre-filtered Winkler BR compared to the in situ BR measured by the in vivo ETS method on the NW Iberian Peninsula shelf and shelf-break, and the North Pacific Subtropical Gyre.

Bacteria are considered major contributors to CR and thus constrain the estimates of both carbon remineralization and biogenic carbon export in the upper ocean (Lemée et al. 2002; Rivkin and Legendre 2001). In some studies, the measured BR accounts for as much as 80%–100% or even greater than 100% of the CR (Biddanda et al. 2001; Kirchman et al. 2009). The unrealistic values of BR% (> 100%) are likely a result of an overestimation of BR due to the methodological artifacts discussed above. The FBR_m_ obtained by the Winkler method contributed to ~ 91% of CR on average in this study, with FBR% higher than 100% observed in six samples. After correction, however, the FBR accounted for an average of just 26% of CR (*n* = 17; 28% at EO and 23% at WE), which is ~ 70% lower than the uncorrected ratio (Fig. 6A, B). These results are consistent with previous reports that have shown overestimation of FBR% by an in vivo electron transport system (ETS) assay. For example, Aranguren-Gassis et al. (2012) reported similar FBR% ratios of ~ 30% in both oligotrophic (*n* = 127) and productive regions (*n* = 19), whereas the ratios obtained by the Winkler method were ~ 155% and ~ 42% in oligotrophic and highly productive areas, respectively. Martínez-García et al. (2013) also showed FBR% of 31% (*n* = 20) in unfiltered seawater by ETS method, while the BR% reached 109% using the in vivo ETS method and 185% using the Winkler method in the pre-filtered 24 h incubated seawater. Interestingly, the mean contribution of FBR to CR is quite stable across different ecosystems.

Enhanced growth in the FL fraction in unfiltered seawater suggests the potential overestimation of CR. A comparison of the CR from the oxygen-based in vitro incubations with the biogeochemical models also suggests an approximately 30% overestimation of measured CR, leading to the identification of the prevailing heterotrophic state in the western Pacific (Huang et al. 2019). Although the exact reason for this overestimation needs to be further explored, our result provides a clue showing that the active growth of bacteria in the whole community could make a certain contribution to the overestimation of CR.

It worth noticing that most particles, including the particle-attached (PA) bacteria, were excluded from all above BR measurements and reports by the prefiltration procedure. PA bacteria densely colonize almost all types of particulate organic matter (Grossart 2010; Simon et al. 2002) with different community composition and metabolic activity from the FL bacteria (Caron et al. 1982; Crump et al. 1998; Simon et al. 2014). Indeed, we determined that the PA bacteria accounted for 11.4% of total bacterial abundance (9.5% at EO and 13.4% at WE) and 47% of total BP (44% at EO and 49% at WE) (Guo et al. in prep.), suggesting that they are a functionally important portion of the total bacteria. Removal of this fraction may lead to a potential underestimation of total BR. Thus, we attempted to estimate the contribution of respiration of PA bacteria to CR. The BR of PA bacteria (PBA) were estimated by the Metabolic Theory of Ecology (MTE) model, which characterizes the effects of cell size and temperature on the metabolism of organisms (Brown et al. 2004). Details of the calculation and discussion are shown in the supplementary materials. We estimated that the BR of PA bacteria contributed ~ 24% to total BR and ~ 8% to CR, which makes it an essential component that cannot be neglected when evaluating total BR in coastal waters of the NW Pacific. The underestimation of PA BR may counterbalance a portion of overestimation of FBR, when estimating the total BR.

### Potential underestimation of BGE

A reliable estimation of BGE is of great importance because it affects the recognition and interpretation of the carbon cycle in microbial food webs and the proportions of carbon in particulate and dissolved forms. Such information is required for building more realistic and reliable models of the carbon cycle in aquatic systems. Previous work has reported mean BGE values of 0.22 in oceans and 0.33 in estuaries (del Giorgio and Cole 1998) and established that BGE may decrease with temperature, implying that bacterial respiration may further exacerbate the effect of ocean warming (Rivkin and Legendre 2001). However, most of the data were obtained from noncomparable measurements of BR and BP, or from long-term experiments monitoring changes in DOC and POC in diluted and filtered seawater of natural bacterial assemblages. BGE calculated by an instantaneous TBP_0_ and an integrated mean FBR_m_ over a 24 h incubation may deviate significantly from in situ values. In this study, the BGE_meas._ calculated with TBP_0_ and FBR_m_ was generally within the same range as in other studies conducted in the same region (Xu et al. 2018). However, by unifying the time and size scales of FBP and FBR, the BGE_corr._ increased significantly by ~ 52% compared to BGE_meas._. Del Giorgio et al. (2011) also reported an underestimation of BGE when comparing different BGE values derived using integrated values of BP over different incubation time scales (0 h, 0–12 h, 0–24 h) and the measured BR in pre-filtered seawaters. The absolute amount of underestimation was also significantly correlated with the difference in gross and net growth rate of FL bacteria (μ_F_fil._- μ_F_unfil_), which can be seen as resulting from grazing mortality (Fig. 5D). This suggests that more of the assimilated organic substrate is used to build up new biomass in bacteria than was previously recognized, and this might be especially the case in eutrophic environments that support a higher bacterial grazing pressure.

### Environmental factors influence the accuracy of BR measurements by the Winkler method

Overall, the inaccuracies of BR measurements were a result of the artifacts created by the experimental procedures of the Winkler method. However, environmental factors may also influence the degree to which BR and BGE are over- or under-estimated. In this study, bacterial metabolic rates were measured at two coastal stations (EO and WE) with very different hydrographic conditions and across two distinct seasons (dry and wet seasons). It was found that FBR_m_ and BGE_meas._ were more biased from the corrected values in the wet season than in the dry season at the eutrophic estuarine station WE, but showed a similar degree of relative change at the more pristine oceanic station EO (Fig. 6C, D). The highest relative changes in FBR_0_ and BGE_corr._ obtained in the wet season at WE were coincident with the significantly higher nutrient and Chl *a* concentrations during the period when the impact of the Pearl River discharge was strongest. The seasonal and spatial variations were likely affected by the degree of simulation of bacterial growth rates in the pre-filtered seawater relative to unfiltered seawater (or, top–down mortality, μ_F_unfil._ − μ_F_fil._), as indicated by the significant correlations of the relative changes of FBR_0_ to FBR_m_ with μ_F_unfil._ − μ_F_fil._ (Fig. 5). Faster bacterial growth is usually associated with higher grazing activity and nutrient concentrations (Kirchman 2016). The close coupling of growth and grazing mortality, which was estimated by difference between gross and net growth rate of cyanobacteria and phytoplankton, has been suggested in many previous studies (Chen et al. 2009; Guo et al. 2014). Indeed, both μ_F_fil._ and μ_F_unfil._ − μ_F_fil._ are significantly correlated with the dissolved inorganic nitrogen concentration (Fig. 5A, B). Thus, the BR estimates by the Winkler method may be more biased in an environment with higher bacterial growth rate and tightly coupled grazing mortality, as well as higher nutrient concentrations. This is probably due to the dominance of “opportunistic copiotrophs” or “r-strategists” bacteria in eutrophic environments, which have larger genomes and cell sizes and can respond quickly to external perturbations, such as relief from grazing pressure and enrichment of nutrients (Lauro et al. 2009). Increases in bacterial cell size after incubation also suggested the outgrowth of larger copiotrophic bacteria. Thus, a more overestimated FBR and FBR% as well as a more under-estimated BGE were observed at WE, with higher nutrient concentrations and bacterial growth rate than those at EO. Notably, since the number of the particle-attached bacteria was higher at WE, the overestimation of FBR could be partially offset by the simultaneous removal of PA bacteria that possess higher individual metabolic rates (supplementary materials).

## Conclusion

In this study, effect of prefiltration and incubation during Winkler BR measurement were assessed in coastal waters of the NW Pacific. Our results showed significant enhancement of FL bacterial abundance, production and cell size during incubation. These experimental artifacts caused significant overestimation of BR measured by the oxygen-consumption Winkler method. The improved estimation of the instantaneous FL BR was ~ 70% lower than that obtained from the Winkler method. The degree of overestimation was mostly affected by the environmental factors of the surrounding seawater. Other carbon metabolic parameters estimated according to BR, such as BGE, were also biased. The corrected BGE with time-integrated free-living BR and BP during 24 h incubation in the pre-filtered sample was 52% higher compared to the common estimations using the noncomparable measurements of integrated free-living BR and instantaneous total BP. Furthermore, the overestimation of BR also exaggerated the contribution of BR to CR. Our results may improve the assessment of carbon dynamics of bacteria in the oceans and raise a warning to be cautious when making estimation of carbon flow through the complex microbial networks in aquatic ecosystems.

## Materials and methods

### Sampling and hydrographical conditions

Monthly samplings were conducted at two subtropical coastal sites, WE (22° 21.32′ N, 113° 56.78′ E) and EO (22° 20.45′ N, 114° 17.70′ E), in the NW Pacific from November 2014 to October 2015 (excluding April, May and June) (Fig. 1). Temperature and salinity were measured during sampling using a YSI 6600 multi-probe sensor. Surface seawater (0 to 3 m) was collected using an acid-cleaned 5 L polycarbonate carboy and transported to the laboratory within 2 h. After seawater arrived in the lab, the samples were immediately processed, and bioassays were conducted to analyze nutrients and measure metabolic rates of bacteria. Samples for dissolved inorganic nutrient analysis were filtered through a GF/F membrane and measured with a SKALAR autoanalyzer following the automated colorimetric technique (Grasshoff et al. 1999). Samples for DOC were filtered through a 0.2 μm filter and analyzed following the high temperature combustion method using a Shimadzu TOC-5000 analyzer (Knap et al. 1996).

### Experimental setup

Subsamples of collected seawater were filtered through 1 μm pore-size, 47 mm polycarbonate membrane filters to obtain the FL bacteria. A low pressure (< 150 mm Hg) was used during the filtration to minimize cell rupture. The unfiltered (original) and 1 μm filtered seawater samples were incubated in eleven replicate, 60 ml biological oxygen demand (BOD) bottles for bioassays. Eight of the BOD bottles were used to analyze BR, and the other three in parallel were used to analyze bacterial abundance, cell size and BP. BOD bottles were filled with seawater by inserting the outflow tube from the carboy into the bottom of the bottle and allowing the seawater to overflow for 4–5 ml. BOD bottles containing seawater samples were incubated in the dark for 24 h in a plastic tank with running seawater to maintain the ambient temperature. Bacterial abundance, cell size and metabolic rates were measured at the beginning (T0) and the end (T24) of the 24 h incubation.

### Bacterial abundance (BA), cell size, growth rate and grazing mortality

Abundance of FL bacteria (FBA) in the 1 μm filtered seawater samples was measured by directly subsampling a 1.8 ml sample from the filtered seawater before and after the 24 h incubation. Abundance of FL bacteria in the unfiltered seawater was measured by filtering seawater samples through a 1-μm polycarbonate filter before collecting the 1.8 ml samples. Triplicate seawater samples in aliquots of 1.8 ml were fixed with 0.5% (final conc.) seawater-buffered paraformaldehyde and stored at -80 °C until analysis. To quantify bacterial abundance, samples were thawed at room temperature, stained with 0.01% SYBR Green I (Invitrogen, CA, USA) and incubated in the dark at 37 °C for ~ 60 min (Marie et al. 1997). Yellowish green fluoresence beads (1 µm, Polysciences Inc., PA, USA) were added as an internal standard to calibrate and normalize the fluoresence and light scattering signals. Analysis was performed with a Becton–Dickinson FACSCalibur flow cytometer equipped with a 488 nm argon laser. The bacterial abundance was determined with WinMDI software 2.9 (Joseph Trotter, Scripps Research Institute, La Jolla, CA, USA), using a bivariate cytogram of side scattering versus green fluorescence, which is used as a proxy for DNA content and cell size. The side-scatter signal was used to estimate cell size (Calvo-Díaz and Morán 2006; Zubkov et al. 1998). Side-scatter units were transformed into cell volume using a size calibration performed with the same cytometer (Chen et al. 2011). Note that, besides cell size, factors, such as shape, cellular inclusions and other physical characters, also affected the side scatter. As such, the cell size obtained from the flow cytometric signal is a rough estimation. In this study, the average equivalent sphere diameter of the bacterial community was 0.5–0.7 μm, corresponding to cell volume of ~ 0.06–0.1μm^3^. These estimates were within a reasonable range of bacterial volume and comparable to other reports (0.054 to 0.122 μm^3^ in Sherr et al. 1997; 0.052–0.195 μm^3^ in Carlson et al. 1999).

Net growth rate of bacteria was calculated using bacterial biovolume-normalized carbon biomass, because bacterial cell size changed (increased in most samples) after the incubation. Biovolumes of bacteria were converted to carbon content (pg C cell^−1^) using a conversion factor of 0.35 pg C/µm^3^ (Bjørnsen 1986). Total biovolume-normalized carbon biomass (μg C/L) was calculated as the product of cellular carbon content and bacterial abundance (cells/L). Net bacterial growth rate (day^−1^) of total (*μ*_T___unfil._) and FL bacteria (*μ*_F___unfil._) in unfiltered seawater, and FL bacteria in 1 μm filtered seawater (*μ*_F_fil._) was estimated by the change of natural logarithm-transformed carbon biomass before and after the 24 h incubation. Grazing mortality of FL bacteria was calculated by the subtraction of *μ*_F_unfil._ from *μ*_F_fil._ (*μ*_F_unfil._ − *μ*_F_fil._).

### Bacterial production

BP of FL bacteria (FBP) were determined at the beginning (0 h) and final time point (24 h) in both pre-filtered and unfiltered seawater samples, by measuring the incorporation rate of ^3^H-Leucine, according to the JGOFS protocol (Knap et al. 1996). In the unfiltered incubations, seawater was filtered through a 1 μm membrane filter and the filtrate was sampled as the FL bacteria to measure FBP. Briefly, 1 ml seawater was added to four replicate 1.5 ml sterile microcentrifuge tubes: three for bioassays and one for the killed control. Seawater samples were incubated with 25 nmol/l ^3^H-leucine for 1 h at ambient temperature. The incubation was terminated by adding trichloroacetic acid to a final concentration of 5%. Trichloroacetic acid was added to the control tube immediately after the addition of ^3^H-leucine. The samples were filtered onto 0.2 μm nitrocellulose membrane filters and washed twice with 5% trichloroacetic acid and then twice with 80% ethanol. Filters containing collected samples were transferred to scintillation vials and 0.5 ml ethyl acetate was added. Tubes were filled with 3 ml liquid scintillation cocktail (Optiphase HiSafe 3, Perkin Elmer) and radioactivity was detected with a Perkin-Elmer Wallac 1414 scintillation counter. BP was calculated from the incorporation rate of ^3^H-leucine using the empirical conversion factor of 3 kg C mol^−1^ leucine (Pedrós-Alió et al. 1999). This conversion factor was obtained from previous studies (Yuan et al. 2011) and has been used to convert leucine incorporation rate to carbon production rate in Hong Kong coastal waters (Xu et al. 2013).

An integrated mean value of FBP (FBP_m_) during the 24 h incubation was computed using the following Eq. (1):

1 $${\mathrm{FBP}}_{\mathrm{m}}=\frac{{\mathrm{FBP}}_{0}({\mathrm{e}}^{\mu t}-1)}{\mu t},$$

where FBP_0_ is the FBP measured at 0 h in the pre-filtered BR incubation bottles, *μ*_._ is the specific growth rate (day^−1^) in the BR bottles in terms of FBP, and *t* is the incubation time (1 day). This equation is derived from models that compute integrated mean values of plankton biomass that change with time by an exponential model in a closed environment (Landry et al. 2003).

### Bacterial respiration by the Winkler method

BR of FL bacteria (FBR) was measured by the Winkler method that quantifies the consumption rate of dissolved oxygen in 1 μm filtered seawater in BOD bottles after a dark incubation. The content of the four replicate BOD bottles was immediately fixed with Winkler reagents to determine the initial oxygen concentration (at 0 h), while the other four replicate BOD bottles were carefully sealed and incubated for 24 h in the dark before fixing, to determine the oxygen concentration after 24 h. The fixed oxygen was titrated using automated titration apparatus (716 DMS Titrino, Metrohm^®^). FBR was calculated from the rate of oxygen consumption from 0 to 24 h and converted to CO_2_ production rate by assuming a respiratory quotient (RQ) of 1 (Robinson, 2008).

BR measured by this Winkler oxygen consumption method, referred as FBR_m_ hereafter, is an integrated mean value of BR of FL bacteria over the 24 h incubation.

### Estimation of BGE

In most studies, BGE was obtained from direct measurements of FBR_m_ and instantaneous total bacterial production at the initial time point (TBP_0_) as Eq. (2),

2 $${\mathrm{BGE}}_{\mathrm{meas}.}= \frac{{\mathrm{TBP}}_{0}}{{\mathrm{TBP}}_{0}+{\mathrm{FBR}}_{\mathrm{m}}},$$

where the TBP_0_ and FBR_m_ were not matched in terms of incubation time and bacterial-size scale. Thus, with integrated mean FBP_m_ and FBR_m_ that measured parallelly in the BR bottles, the corrected estimates of BGE were calculated as Eq. (3):

3 $${\mathrm{BGE}}_{\mathrm{corr}.}= \frac{{\mathrm{FBP}}_{\mathrm{m}}}{{\mathrm{FBP}}_{\mathrm{m}}+{\mathrm{FBR}}_{\mathrm{m}}}.$$

### Correction of BR

Given that the filtration and incubation procedures of the oxygen consumption method may create experimental artifacts, as described in the introduction, the directly measured FBR_m_ may represent a biased rate that differs from the real BR.

Unlike the short incubation time (1 h) applied in BP measurement that minimizes the growth of FL bacteria in filtered seawater, assay incubation time for BR measurement cannot be shortened to a comparable time scale of ~ 1 h due to the sensitivity of the oxygen consumption method. Instead, the instantaneous bacterial respiration rate of FL bacteria (FBR_0_) that matches FBP_0_ on the same measurement time scale can be calculated by the following Eq. (2) assuming BGE remained constant during the incubation.

4 $${\mathrm{FBR}}_{0}=\frac{{\mathrm{FBP}}_{0}}{{\mathrm{BGE}}_{\mathrm{corr}.}}-{\mathrm{FBP}}_{0}.$$

All parameters of bacterial metabolism used in this study and their abbreviations are summarized in Table 1.

**Table 1:** Abbreviations of bacterial metabolic parameters used in this study

Abbreviation	Description
*μ*_F_fil_	Growth rate of free-living bacteria in 1 μm pre-filtered seawater
*μ*_T_unfil_	Growth rate of bacteria in unfiltered seawater
*μ*_F_unfil_	Growth rate of free-living bacteria (< 1 μm) in unfiltered seawater
FBP_0_	Instantaneous production rate of free-living bacteria measured at the beginning of incubation
TBP_0_	Instantaneous production rate of total bacteria measured at the beginning of incubation
FBP_m_	Integrated mean production rate of free-living bacteria during 24 h incubation
FBR_0_	Instantaneous respiration rate of free-living bacteria at the beginning of incubation
FBR_m_	Integrated mean respiration rate of free-living bacteria during 24 h incubation (Bacterial respiration rate measured by the Winkler O_2_ consumption method)
BGE_meas_	Bacterial growth efficiency calculated by TBP_0_ and FBR_m_
BGE_corr_	Bacterial growth efficiency calculated by FBP_m_ and FBR_m_

## Supplementary Information

Below is the link to the electronic supplementary material.

Supplementary file1 (DOCX 327 KB)

## Data Availability

All data generated or analyzed during this study are included in the manuscript and supporting files.
