# Supplementary material for: Making comparable measurements of bacterial respiration and production in the subtropical coastal waters
Source: Mar Life Sci Technol. 2022 Jul 11;4(3):414–27. doi: 10.1007/s42995-022-00133-2 (PMC10077172; doi:10.1007/s42995-022-00133-2)
Supplement: Supplementary file 1 — Supplementary file1 (DOCX 327 KB) [file 42995_2022_133_MOESM1_ESM.docx]

Supplementary materials

**Experimental Problems in the Bacterial Respiration Rate Measurement Based on Oxygen-Consumption Involving Prefiltration and Incubation**

Fig S1. Bacterial growth curves (solid line) of representative samples at (A) EO and (B) WE. The dashed lines are linear regression of the exponential growth phase.

**Estimation of respiration of particle-attached bacteria (ABR)**

BR of PA bacteria (ABR) cannot be directly measured in the field samples because of the similar size ranges shared by PA bacteria and some phyto- and zoo-plankton that make it impossible to physically separate them and measure their respiration rate experimentally. An alternative way is to estimate metabolic rates of organism by the Metabolic Theory of Ecology (MTE) model, which characterizes the effects of cell size and temperature on the metabolism of organisms (Brown et al., 2004; López-Urrutia and Morán, 2007). We assumed that the cell specific respiration rate (sBR) of both the FL and PA fractions follows the same scaling law governed by the MTE model, which is

$\ln\left( \mathrm{sBR} \right)=\alpha\ln V-E\left( \frac{1}{\mathrm{kT}} \right)+b$ (S1)

where V is cell volume (μm^3^), T is the absolute temperature (K), k is the Boltzmann constant (1.38 × 10^-23^ J/K), α and E are scaling exponents for cell size and temperature, and b is a normalizing constant. The α value of 1.7 was used as a scaling exponent for cell size according to Delong et al. (2010) and García et al. (2016), while the values of E and b were determined from linear regression analysis of [ln(sBR_F_)-1.7lnV] with the temperature function (1/kT) in this study. The sFBR was calculated by dividing the corrected FBR_0_ (μg C L^-1^ d^-1^) by cell density of free-living bacteria (cells L^-1^).

Linear regression analysis of the natural logarithm of sFBR_0_ after subtracting the effect of cell volume (ln sBR_F_-1.7 ln V) with the temperature function, 1/kT, showed a significant temperature dependence of sFBR. A temperature scaling exponent of 0.817 eV and a normalizing constant b of 40.089 were obtained, and the following Equation (S2) was obtained to calculate cell-specific particle-attached BR (sPBR).

$\ln\left( \mathrm{sBR} \right)=1.7\ln V-0.817\left( \frac{1}{kT} \right)+40.089$ (S2)

The temperature dependence of sFBR_0_, which represents the activation energy or thermal sensitivity of the bacterial metabolism, is ~0.817 eV. This value is higher than the activation energy of ~0.65 eV (1.602×10^-19^ J) as reported for general heterotrophic organisms (Gillooly et al. 2001; Brown et al. 2004; Allen et al. 2005) but similar to a recently published mean value of ~0.88 eV for bacteria and archaea, suggesting prokaryotic metabolisms are probably more sensitive to temperature than that of eukaryotes (Smith et al. 2019). By scaling sFBR_0_ with the cell volume, we obtained a scaling exponent (α) of 1.805 (data not shown). This is consistent with previous estimations on the scaling exponent (1.6-1.8) of prokaryotes in marine environments (DeLong et al. 2010; García et al. 2016). A scaling exponent (α) of 1.7 was chosen to estimate the effect of cell size on BR in this study, according to DeLong et al. (2010) who reported an α of 1.7–2.0 from a compilation of data for terrestrial and aquatic prokaryotes, and García et al. (2016) who reported an α of 1.67 from sBR scaling with cell size along a latitudinal transect in the Atlantic Ocean.

sPBR ranged from 19 to 117 pg C cell^-1^ d^-1^ with a mean value of 49 pg C cell^-1^ d^-1^ at the two stations. Compared with sFBR, sPBR was overall ~4-fold higher (paired t-test, n=17, *P*<0.01). PBR of the whole PA bacterial community ranged from 1.3 to 38 μg C L^-1^ d^-1^ with a mean value of 10 μg C L^-1^ d^-1^, 3.5-fold lower than FBR. Overall, PA bacteria contributed 24% to total BR and 8% to community respiration (CR) in coastal waters of the NW Pacific.

Reference:

DeLong JP, Okie JG, Moses MF, Sibly RM, Brown JH (2010) Shifts in metabolic scaling, production, and efficiency across major evolutionary transitions of life. Proc Natl Acad Sci USA 107: 12941-12945

García FC, García-Martín EE, Taboada FG, Sal S, Serret P, López-Urrutia A (2016) The allometry of the smallest: superlinear scaling of microbial metabolic rates in the Atlantic Ocean. ISME J 10: 1029-1036

Smith TP, Thomas TJ, García-Carreras B, Sal S, Yvon-Durocher G, Bell T, Pawar S (2019) Community-level respiration of prokaryotic microbes may rise with global warming. Nat Commun 10: 1-11
